# 

**Published:** 1833

**Authors:** 


I.—Epidemic Cholera: Miscellaneous Observations—historical, statistical, ætiological and therapeutic, on the prevailing Epidemic. By Daniel Drake, M. D. - 161

1. Spread of the Epidemic, - - - - - - 162
2. Ætiological Remarks, - - - - - - 163
3. Exciting Cause, - - - - - - - - 168
4. Treatment of the Epidemic in Lexington, - - 169
5. Cholera in Cincinnati, - - - - - - 172
6. Fatal Cases, - - - - - - - - - 174

II. Anomalous and Fatal Case of Disease. By A. W. Bowen, 182

### Reviews and Bibliographical Notices.

III. Diseases of Louisiana. The application of Physiological Medicine to the Diseases of Louisiana. By Edward H. Barton, M. D., - - - - - - 187

IV. Human Physiology. Human Physology Illustrated by numerous Engravings. By Robley Dunglisson, M. D., 193

V. Physical Education. Remarks on the Influence of Mental Cultivation upon Health. By Amariah Brigham.

First Annual Report of the Society for promoting Manual Labor in Literary Institutions, including a Report of their General Agent. By Theodore D. Weld, - 226

1. Absurdities and Excesses in Diet, - - - 228
2. Abuse of Stimulating Drinks, - - - 229
3. " Tobacco, - - - - - - 230
4. Absurdities in Dress, - - - - - - ib
5. Irregularities in Sleep, - - - - - 232
6. Immersion in a Confined Atmosphere, ib
7. Neglect of Exercise, - - - - - - 233

### Miscellaneous Intelligence.

### ANALECTIC.

1. Observations on Cold as a Cause of Disease. - - 256
2. On Muscular Contractions, and Animal Electricity, - 263
3. Treatment of Inflammation of the Lungs, - - - - 265
4. Gonorrhœa and its Treatment, - - - - - - 266
5. The Anodyne Metallic or Galvanic Brush, - - - - 268
6. Origin of Acephalocysts, - - - - - - - - 270

7. Internal Use of Chlorine in Nervous Fever, - - 271
8. Endemic Therapeutics, - - - - - - ib
9. Mode of Dressing a Stump after Amputation, - - - ib
10. Changes which the Points of the Fingers undergo in Phthisis, 272
11. Ingenious method of applying Nitras Argenti to the Ulcers of the Cornea, - - - - - - - - - 273
12. Roseolous Eruption, caused by the use of Copaiba and Cubebs, - - - - - . - - - ib
13. New Theory of Sounds of the Heart, - - - 274
14. Ergot. By Dr. Borllcher, - - - - - - - ib
15. On Ergot, M. M. Trousseau, - - - - - - 275
16. Chemical Analysis of Ergot, - - - - - - 276
17. Anthelmintic Emulsion, - - - - - - - - 277
18. Infantile Convulsions, - - - - - - - ib
19. Employment of Chloride of Lime in the Treatment of Psora, - - - - - - - - - - 281
20. Chronic Bronchitis, - - - - - - - - 282
21. Method of applying Nitrate of Silver to Ulcers of the Cornea, - - - - - - - - - 283
22. Chloride of Soda in Burns, Scalds, and Black Eyes, - ib
23. Prolapsus Ani, - - - - - - - - - 284
24. On the Use of Cold Water in Cholera, - - - 286
25. Use of Ice and Chloride of Soda in Cynanche Maligna. - 290
26. Efficacy of the Cold Dash in Nervous and Convulsive Diseases, - - - - - - - - - 292
27. On the Formation of Callous, and the mode of Remedying it when diseased or deformed, - - - - 293
28. Gangrene of the Mouth in Children, - - - - 295

### ANALYTICAL.

1. Puerperal Fever, - - - - - - - - 297
2. Spinal Origin of Rheumatism, - - - - - 305
3. Electro Therapeutics, - - - - - - - 309
4. Dysmenorrhœa, - - - - - - - - 313

### ORIGINAL.

1. Our Second Hexade, - - - - - - - 316
2. Extra Professional Duties, - - - - - - ib
3. Medical Journal on a New Plan, - - - - - 318
4. Geological Survey of the State of Tennessee, - 319
5. Flora of Kentucky. - - - - - - - - ib
6. Cabinet of Entomology For Sale, - - - - - ib
7. Mortification and Sloughing of a part of the Intestine in Hernia. Recovery without an External Aperture, - 320
8. Appointments in the Medical College of Ohio, - - ib

